# Launching Austria’s One Health network: paving the way for transdisciplinary collaborations

**DOI:** 10.1186/s42522-024-00116-6

**Published:** 2024-10-28

**Authors:** Amélie Desvars-Larrive, Pamela Burger, Johannes Lorenz Khol, Annika Posautz, Eva Schernhammer, Ruth Kutalek, Gavrila Amadea Puspitarani, Lia Schlippe Justicia, David Niklas Springer, Damien Ernst, Cynthia Sohm, Anna Pontel de Almeida, Hermann Schobesberger, Sascha Knauf, Chris Walzer

**Affiliations:** 1 Clinical Department for Farm Animals and Food System Science, University of Veterinary Medicine Vienna, Veterinaerplatz 1, Vienna, 1210 Austria; 2 Complexity Science Hub, Josefstaedter Strasse, Vienna, 1080 Austria; 3 Department of Interdisciplinary Life Sciences, University of Veterinary Medicine Vienna, Savoyenstraße 1, Vienna, 1160 Austria; 4 Vice-Rectorate for Study Affairs and Clinical Veterinary Medicine, University of Veterinary Medicine Vienna, Veterinaerplatz 1, Vienna, 1210 Austria; 5 Department of Epidemiology, Medical University of Vienna, Kinderspitalgasse 15, Vienna, 1090 Austria; 6 Department of Social and Preventive Medicine, Medical University of Vienna, Kinderspitalgasse 15, Vienna, 1090 Austria; 7 Center for Virology, Medical University of Vienna, Kinderspitalgasse 15, Vienna, 1090 Austria; 8 Department of Biological Sciences and Pathobiology, University of Veterinary Medicine Vienna, Veterinaerplatz 1, Vienna, 1210 Austria; 9 Institute of International Animal Health/One Health, Friedrich-Loeffler-Institut, Federal Research Institute for Animal Health, Südufer 10, Greifswald - Insel Riems, 17493 Germany; 10 International Animal Health, Faculty of Veterinary Medicine, Justus Liebig University, Frankfurter Strasse 106, Giessen, 35392 Germany; 11 Wildlife Conservation Society, 2300 Southern Boulevard Bronx, New York, 10460 NY USA

**Keywords:** One Health, Network, Austria, Workshop

## Abstract

In the post-COVID-19 era, stakeholders, including policymakers, funders, and the public, are increasingly seeking for a cross-sectoral systems-based approach to health risks extending beyond conventional measures. Anchored on three health pillars -human, animal, and environmental- One Health offers a promising framework to effectively address this demand. While some nations have already implemented national One Health strategic plans, European countries, in general, are lagging behind the global agenda. On 22 February 2024, an initiative was launched in Austria toward addressing this gap, bringing together multiple sectors and disciplines, marking the initial step in creating a national One Health network. The workshop emphasized the importance of enhancing One Health education and addressed key topics, such as incorporating the environmental pillar of One Health as well as socio-economic and cultural drivers to further our understanding of outbreaks, and establishing trusted communication channels, including data sharing, between disciplines and sectors. Identified challenges encompassed the need for more funding of transdisciplinary research. Opportunities for advancement include initiating local One Health projects and showcasing their positive impacts. Moving forward, efforts will focus on establishing a mature and globally connected One Health framework in Austria and supporting the integration of One Health aspects into education curricula, research programs, and policies.

## Background

As we navigate the landscape of polycrisis in the post-COVID-19 pandemic period and prepare for the next disease outbreak, governments, policymakers, multilaterals, donor organizations, and civil society demand a more comprehensive approach to the health-environment-climate nexus. An expanded systems-based approach to health, as outlined in the One Health framing [1], requires clear hazard identification paired with equitable risk management that extends far beyond conventional public health measures. Multiple initiatives are emerging in this rapidly evolving space, bringing together scientists, stakeholders, and community groups from diverse disciplines, fields, and sectors [2, 3]. Establishing collaborative networks that enhance communication, foster political commitments, and drive scientific advancements is a pivotal initial step. Here, we distinguish between different types of collaboration: “interdisciplinary” brings together experts from different fields to create a unified approach and involves combining knowledge and methods to solve complex problems [4]; “transdisciplinary” is defined by the inclusion of non-academic stakeholders in the process of knowledge production [5] while “intersectoral”, “multisectoral”, and “cross-sectoral” refer to collaboration with one or more government sectors [6].

Lacking a central, independent, coordinating One Health entity, Austria’s assessment of health hazards and management of health risks and research between various ministries -such as agriculture, forestry, human and animal health, research funding, climate, and biodiversity- remains fragmented and creates durable barriers to synergistic action. This situation is compounded by the fact that, in contrast to animal health, human health remains the remit of the individual EU member states, which limits powers to enforce a common health strategy and has led to fragmentation in responses to, for example, COVID-19 [7, 8].

Currently in Austria, health professionals and researchers often operate in relative isolation, forming collaborations on an ad-hoc basis [9] and struggling to achieve critical mass. While in Europe, including Austria, the One Health approach is often focused on antimicrobial resistance (AMR) and zoonotic diseases, its broader implementation remains limited [10]. There are several opportunities for improvement in the Austria’s approach to achieve One Health, including streamlining bureaucratic processes, enhancing communication between the human, animal and environmental sectors, and optimizing resource allocation. Addressing these areas is a prerequisite for the necessary transformation process that will ultimately lead to more cohesive and effective health practices.

For instance, despite relatively low levels of AMR in Austria [11], and while the country is already implementing a human-animal approach to this issue [12], several factors currently hinder effective evidence-based management of AMR. These include policy fragmentation, dispersed responsibilities, neglecting the environmental compartment, prioritization of agenda conformity over AMR mitigation measures, lack of appropriate recognition for stewardship, fragmented data, and non-interoperable systems [13]. These challenges also hamper precise epidemiological evaluation and risk analysis [14]. Forming a transdisciplinary and cross-sectoral advisory group of professionals could address this “implementation gap” by providing the necessary coordination and expertise.

A recent study highlights the difficulties associated with scattered data on zoonotic agents and their sources, emphasizing the urgent need for a more unified approach to zoonotic risk assessment and prevention [15]. The emergence of zoonotic pathogens, with at least eight new agents identified in Austria over the past 20 years [15], alongside the increasing incidence of infections like leishmaniasis [16] and tularemia [17], and the increasing zoonotic risk posed at the livestock-human, food-human, wildlife-human, and wildlife-livestock interfaces [15, 18–20] further underscores this necessity.

Although Austria is officially free of zoonotic tuberculosis (TB), the western part of the country has experienced sporadic TB cases in cattle over the past decade, due to co-grazing with the maintenance host of *Mycobacterium tuberculosis* variant *caprae*, the red deer, on alpine pastures. The infection dynamics remain poorly understood [19, 20], notably, whether environmental persistence and transmission might play a role [21]. The transboundary nature of these interconnected challenges (i.e., biodiversity, livestock management, and disease transmission), exacerbated by divergent conservation and hunting regulations among and within European Alpine countries [22], and the lack of coordinated health responses, hinder effective national and cross-border eradication efforts. Similarly, the spatial distribution of the phylogenetic clusters of Puumala orthohantavirus (PUUV) found in human patients in Austria is influenced by host-pathogen evolutionary mechanisms within the enzootic host, the bank vole, *Clethrionomys glareolus*, which geographic distribution is driven by habitat and climate suitability, and is affected by potential ecological barriers to population movements [23, 24]. Notably, the “Alpe-Adria” genotypes found in both humans and rodents in southeastern Austria extend to the neighbouring countries, Slovenia and Hungary [23].

Finally, in Austrian educational curricula, a crucial need remains to actively integrate the One Health concept. While it receives some support and advocacy within veterinary training programs, its representation in other fields, such as medical universities, is particularly lacking and must be significantly improved.

## The workshop

On 22 February 2024, the University of Veterinary Medicine Vienna, Austria, invited an interdisciplinary group of interested scientists from diverse universities, and representatives from key institutions, including the Ministry of Education, Science and Research, the Ministry of Agriculture, Regions and Tourism, national funding agencies, the National Public Health Institute, and the Austrian Agency for Health and Food Safety, to collaborate on the establishment of a One Health network in Austria. The initiative received extensive positive feedback and marked a significant milestone in Austria’s commitment towards a unified approach to tackling complex health challenges. This inaugural workshop aimed to provide an opportunity for scientists working in human, animal, and environmental health, as well as conservation, social sciences, and disaster reduction, to come together and discuss the need for and strategies to establishing a One Health network. The workshop involved 39 participants from 21 different Austrian institutions. The event featured keynote speakers, who covered various perspectives of the One Health approach, spanning from global-scale pandemic prevention and preparedness efforts to local investigations of communities affected by disease outbreaks. Additionally, the workshop facilitated two panel discussions and actively engaged participants in gathering ideas and feedback through a World Café session, fostering collaborative dialogue and knowledge exchange [25].

### Keynote address and its resonance with global One Health priorities

The opening remarks underscored that collaboration across sectors and disciplines should strive for far-reaching impacts by embracing a systems-based transformation framework, at local and global levels. Restructuring well-established conservative systems, with entrenched disciplinary specialists, is generally challenging, and transitioning from competition to collaboration necessitates clear (common) goals and incentives.

Operationalizing One Health requires embracing the “4Cs”: Communication, Coordination, Collaboration, and Capacity building [1]. In an era marked by misinformation, disinformation, and gaslighting, One Health must develop a robust ontological basis with a common language across disciplines. Additionally, effective communication must respect customs and cross-cultural differences at the institutional, national, and international scale, respecting context-specific values and resource availability, while avoiding creating an academic One Health silo. Capacity building is crucial for One Health, not only in the Global South, where some countries have already established National One Health Strategic Plans [26], but also in countries of the Global North, where persisting legislative, budgetary, and structural constraints hinder whole-of-government and whole-of-society cooperation. Multi-scale coordination efforts are required for the context-specific design and implementation of One Health interventions, with technical working groups at regional and local levels providing support to navigate across existing silos. Additionally, metrics must be developed to evaluate and validate One Health interventions, allowing for the demonstration of their impact and facilitating efficient use of available resources.

The keynotes also highlighted the significance of anthropological perspectives in understanding the social, cultural, behavioural, and economic dimensions of disease emergence and transmission [27]. These dimensions are essential for contextualizing disease outbreaks, which are frequently considered as “complex systemic challenges” [28], requiring more than just biomedical solutions. One Health approaches in medical anthropology, such as in multispecies ethnography, seek to acknowledge *“the interconnectedness and inseparability of humans and other life forms”* [29], prioritizing the empowerment and inclusion of local communities and standpoints. Notably, documenting people’s narratives offers valuable insights into both the epidemiological aspects and social determinants of health, including health inequalities, surrounding the emergence of infectious diseases. It allows to understand local health practices, risk behaviours, and animal-human-environment relationships.

### Panel discussions: sharing perspectives on One Health

The first panel involved young scientists, specifically Ph.D. students, representing different disciplines and cultural backgrounds. This panel offered an opportunity for young voices to discuss and share their vision of One Health, acknowledging them as the next generation experts. The second panel involved senior scientists actively engaged across health sectors, One Health initiatives, and education.

Discussions in both panels highlighted the necessity for enhanced One Health education, advocating for its incorporation into the curriculum from elementary school through university, enabling students to develop interdisciplinary and systems-thinking skills from an early stage. The young panelists reported the absence of formal One Health training in their academic journeys, emphasizing the persistence of disciplinary silos in education. Additionally, the panelists shared their experiences of seeking out complementary education on One Health after graduating, underscoring the gap in formal training and the importance of self-directed learning in acquiring One Health expertise. Despite initial efforts, such as the establishment of the One Health Doctoral College at the University of Veterinary Medicine Vienna, Austria, and the MYCOS Doctoral Programme on antimycotic resistance launched jointly by the University of Innsbruck and the Medical University of Innsbruck, Austria, these initiatives remain localized and uncoordinated.

All panelists highlighted the importance of fostering trusted communication among disciplines and sectors, particularly underscoring the critical role of data sharing in advancing One Health objectives. Integration of multi-source data stands as a pivotal step in achieving systems-based approach. However, data sharing meets diverse challenges, including ethical, legal, institutional, and occasionally political constraints. Additionally, data integration may be limited by technical obstacles, such as, data quality, granularity, coverage, or format compatibility. The future of One Health in Austria relies on overcoming these data-related barriers and devising sustainable, ethical, and legally sound solutions to break data silos.

The panelists also emphasized the challenges of translating scientific outcomes into actionable policies, highlighting a significant gap between scientific findings and policy uptake, noting that scientists may lack training in effectively communicating with policymakers, while politicians may face difficulties in deciphering data-driven policies. Investing in science-policy interfaces, promoting dialogue between researchers and decision-makers, and raising political awareness can help bridge the gap between One Health scientific knowledge and policy implementation.

They also identified barriers such as resistance to innovative thinking, particularly regarding curriculum changes, persistent disciplinary silos, and limitations in bridging fields due to a lack in relevant professional connections or practical constraints. For instance, non-medical researchers expressed uncertainty about navigating ethical approval processes for community surveys on environmental issues. Additionally, academic incentive systems often discourage collaboration across disciplines, further complicating One Health efforts.

### Participants’ feedback and community engagement: World Café

Three facilitators moderated three parallel discussions during the World Café [25]. Attendees were split evenly into three groups and rotated between each table; each session was built iteratively on the discussions and ideas of the previous groups. At the end, the facilitators summarized the sessions. The World Café was centered on three major questions:

- What are the priorities and challenges for achieving One Health in Austria, including societal dimensions?
- What are potential barriers/gaps and opportunities for collaborative activities in Austria?
- How can we effectively implement a systems-based approach to health in Austria?

#### Priorities and challenges for One Health in Austria

In addressing the priorities for One Health in Austria, the World Café discussions emphasized the importance of government commitment and engagement in establishing a sustainable long-term One Health vision and mission statement, particularly given that mandates are typically short-term. Participants highlighted the need for a stable body to guide and follow up on One Health questions. Additionally, Austria’s high degree of federalism, which enhances fragmentation and parallelism, was identified as a barrier to operationalizing One Health at the national level. Moreover, participants highlighted challenges such as inadequate funding and shortage of suitable reviewers for interdisciplinary research, struggles in identifying common ground across multidisciplinary teams, and a lack of cooperation between academia and industries. Notably, participants observed that crises, such as the Chernobyl disaster and the COVID-19 pandemic, have historically served as catalysts for new funding opportunities and heightened interest in collaborative transdisciplinary work. However, outside of crisis scenarios, proactive investment and forward-thinking initiatives have remained limited. It is imperative not only to capitalize on the increased post-disaster/post-pandemic funding but also to strategize for the gap-phase where funding may be low. Furthermore, it is essential to sensitize funders and governments to the critical importance of prevention. When efficient, prevention can offer long-term benefits such as improved public health outcomes, reduced economic burden, and enhance societal resilience [30].

#### Opportunities for transdisciplinary collaborations

Advancing One Health in Austria necessitates a tiered approach, initiating local One Health projects, and subsequently scaling nationally and internationally. Furthermore, the participants recognized the need to showcase the economic benefits of One Health approaches and policies as a crucial factor in persuading government partners, stakeholders, scientists, and civil society. Identified key priorities included improving communication between political entities and scientists to increase the accessibility of the One Health concept to diverse communities and the general public. Participants also highlighted the importance of incentivizing interdisciplinary cooperation in academia, suggesting including One Health activities in tenure tracks. Funding agencies and donors can contribute to this transition by tendering grants for cross-sectoral and transdisciplinary research. In the long term, a change in the education system, where a confrontation with One Health happens at an early stage (e.g., primary school), should be anticipated. Furthermore, participants proposed implementing a Training of Trainers (ToT) model to reach a broader audience and establish a more sustainable system of One Health training. Participants underscored the critical need to integrate environmental considerations into the One Health framework, demanding their inclusion in future discussions and initiatives. Finally, participants highlighted the importance of expanding the One Health framing beyond the prevalent focus on infectious diseases, e.g., to non-communicable diseases, food security and safety, while incorporating operators of the agri-food sector and economists in future workshops.

#### Systems-based approach to health

To effectively develop and implement a systems-based approach [31] to health in Austria, key strategies will involve an initial stakeholder mapping to identify individuals and institutions addressing health across sectors, including government agencies. Civil society can be engaged through various means, such as citizen science initiatives, to ensure broader community participation and input. Participants emphasized that implementing a systems-based approach demands a fundamental shift in collaboration and budgeting, guided by clear goal definitions and establishing a national One Health mission. Notably, the participants considered developing a concise and comprehensive vision and action plan as essential. For such a process, the guidelines to implement a One Health Joint Plan of Action as developed by the Quadripartite [32] could be adopted. Additionally, the participants underscored that a systems-based approach requires continuous and active engagement across diverse disciplines, sectors, institutions, and actors, adopting a solution-oriented mindset.

## Operational model and vision for the future

The Austrian One Health network will primarily operate as an informal national network, offering flexibility and adaptability to address the dynamic nature of complex health challenges. This model aligns with Austria’s existing institutional frameworks, where a centralized response demonstrated efficiency and high trust in the government during the first wave of the COVID-19 pandemic [33, 34]. In each of the sectors -human, animal and environmental health- Austria has a heavily centralized governance, which involves top-level decision-making with decentralized implementation. Notably, the country ensures universal health insurance for everyone, primarily through the Austrian Health Insurance Fund (Österreichische Gesundheitskasse). To achieve nationwide health capacity, the system relies on decentralized national reference centers and laboratories (see https://www.ages.at/en/ages/locations) as well as health services for both humans (e.g., State Health Directorate [Landessanitätsdirektion]) and animals (State Veterinary Directorate [Landesveterinärdirektion]). By leveraging both centralized coordination and decentralized capabilities, together with an informal network of professionals that facilitates communication, data sharing, and collaborative efforts across different governance levels, sectors, and disciplines, Austria has a unique opportunity for strengthening its One Health surveillance strategy and improving the nation’s overall resilience to emerging health threats.

In the next phase, a One Health platform could be developed as a collaborative digital system, supporting both centralized and decentralized actions. This platform could feature tools for data collection, analysis, and sharing, alongside resources for communication, coordination, and real-time collaboration among stakeholders, further advancing the execution of One Health initiatives. By offering public access to relevant information, the platform will boost the visibility and impact of One Health efforts in Austria. For example, the National One Health Platform in Germany (https://onehealthplatform.net) [35] could serve as a blueprint, but tailored to the specific needs of Austria.

In the long term, we envision a dedicated physical knowledge space, or “One Health hub”, designed to be the central point for transdisciplinary collaboration, innovation, education, and project development. Beyond serving as a physical space, this hub would ideally include dedicated individuals to manage and coordinate the network’s activities. This hub would also bridge science-policy and science-disease management efforts. It would offer the necessary infrastructure and resources to operationalize the One Health network by providing a venue for meetings, workshops, training sessions, and collaborative research projects, thereby facilitating and enhancing the network’s activities. Together, the One Health network, platform, and hub will form a cohesive structure to achieve our vision for comprehensive, integrative health solutions.

## Key challenges in establishing a functioning One Health network

We see several potential limitations in the growth and practical successes of the One Health network in Austria. The network’s activities will require a clear strategic direction and effective coordination to avoid fragmentation and ensure that efforts are aligned with overarching goals. Without these elements, there is a risk of inefficient use of resources, lack of cohesive action, and diminished impact on addressing complex health challenges. The overall strategic impact of the One Health network could be strengthened by adopting clear and transparent reporting practices for its goals and outcomes [2]. Another potential limitation is the insufficient representation of non-academic stakeholders, inadequate representation of the environmental sector [36], and lack of social diversity [2, 37]. Truly mainstreaming One Health across all levels of society requires integrating diverse voices, perspectives, and expertise, essential for an innovative One Health approach. Furthermore, ambiguous accountability structures within the network can lead to unclear roles and responsibilities [2], making it challenging to monitor actions and outcomes. Establishing well-defined roles is crucial for ensuring the network’s efficiency and impacts.

Incorporating collaborative, cross-sectoral One Health governance into Austria’s deeply rooted hierarchical institutional landscape will be challenging, particularly given the fragmentation and complexities of agencies across the European Union, federal government, and individual federal states, each with differing jurisdictional mandates, responsibilities, and varying terms. The presence of organizational silos, where each institution adheres to specific working practices and methods, adds another layer of challenge. To fully realize the potential of One Health, Austria must strengthen communication, coordination, and cooperation strategies that maintain explicit functions and responsibilities while preserving existing command and control systems [38, 39].

Finally, securing sustainable funding will be essential to maintaining the network’s activities and supporting its long-term goals.

## Conclusion

This initial effort in Austria aligns with the broader global One Health agenda [40–42]. Recognizing the imperative to actively, holistically, and collectively address local, national, and international health challenges, Austria urgently needs to structure and strengthen a mature, functional One Health network. The recent workshop, which gathered insights from participants and key stakeholders, lays the foundation for establishing such a network. Moving forward, the proposed actions include mapping stakeholders who could engage in One Health, enlarging the national One Health network, defining clear goals and objectives for Austria’s One Health network, creating a Theory of Change, and identifying potential challenges and gaps while supporting future transdisciplinary collaborations and research initiatives. The overarching goal is to enhance Austria’s capacity to navigate current and future complex health issues across various scales, fostering national resilience and preparedness in the face of ever-increasing One Health challenges. The envisioned One Health network also aims to avoid duplication of efforts by promoting awareness of other initiatives. Furthermore, the network may evolve into an advisory body, offering support for government decision-making. As Austria establishes its One Health network, it could support similar emerging initiatives in Central and Eastern Europe and subsequently merge into a regional cross-border One Health network.

## Data Availability

Not applicable.
